# Hearing Loss: Reestablish the Neural Plasticity in Regenerated Spiral Ganglion Neurons and Sensory Hair Cells 2018

**DOI:** 10.1155/2018/4759135

**Published:** 2018-12-17

**Authors:** Renjie Chai, Geng-Lin Li, Jian Wang, Hai Huang

**Affiliations:** ^1^MOE Key Laboratory of Developmental Genes and Human Disease, Institute of Life Sciences, Southeast University, Nanjing 210096, China; ^2^Co-Innovation Center of Neuroregeneration, Nantong University, Nantong 226001, China; ^3^Biology Department, University of Massachusetts Amherst, Amherst, MA 01003, USA; ^4^School of Communication Science & Disorders, Dalhousie University, 5850 College Street, Halifax, NS, Canada B3J1Y6; ^5^Department of Cell and Molecular Biology, Tulane University, New Orleans, LA 70118, USA

Hearing loss is a common sensory disorder that has been a serious concern globally. It is recently estimated that around 466 million people worldwide have disabling hearing loss, including 34 million children, and this number will increase to over 900 million by 2050 (according to WHO report 2018). The majority of hearing disorders occur due to the death of either inner ear hair cells (HCs) or spiral ganglion neurons (SGNs), thus leading to sensorial neural hearing loss (SNHL). SNHL is known as the most common form of hearing disorder that comprises about 85% of all hearing loss cases. This type of damage is induced by a variety of reasons such as inner ear trauma, ischemia, ototoxic drugs, noise exposure, inflammation, viral infections, genetic deficits, autoimmunologic reaction, and aging. SNHL is generally not reversible due to the lack of regeneration capacity of HCs and SGNs. However, various recent studies have determined that the HCs and SGNs hold a regenerative potential and it is possible to find the cure for SNHL in the near future. This is supported by the clear understanding of the genetical control and signaling pathways involved in the development of HCs and SGNs and their functions, the regenerative potential of residing adult stem cells, and the development of gene therapy and the clinical trials of new pharmaceutical compounds on damaged cochleae. Last year, we have published the first special issue of “Hearing Loss: Reestablish the Neural Plasticity in Regenerated Spiral Ganglion Neurons and Sensory Hair Cells”, and this year in this second special issue, we are presenting a new series of articles to report the most recent advances in several major areas as summarized below: HC development, HC damage and protection, HC regeneration, SGN development and protection, inherited hearing loss, and inner ear drug delivery.

## 1. Hair Cell Development

A. Chang et al. (“Specific influences of early acoustic environments on cochlear HCs in postnatal mice”) report that the early environmental sounds promote functional maturation of HCs. Acoustic environment significantly decreases the ABR threshold, increases prestin expression in outer HCs, and enhance maturation of ribbon synapses in the postnatal mouse cochleae. P. Chen et al. (“Postnatal Development of Microglial like Cells in Mouse Cochlea”) demonstrate that the microglial-like cells are present in the developing mouse cochlea, and these cells go through the drastic morphological and distributional changes during the postnatal cochlear development. Also, these cells might participate in the maturation and remodeling of the cochlea. F. Chen et al. (“Hydromechanical Structure of the Cochlea Supports the Backward Traveling Wave in the Cochlea *In Vivo*”) for the first time experimentally demonstrate the backward traveling wave theory by measuring the phase spectra of the basilar membrane vibration at multiple longitudinal locations along the cochlea. X. Cheng et al. (“Modulation of Glucose Takeup by Glucose Transport on the Isolated OHCs”) report that glucose is transported into OHCs via glucose transporter 1 and 4, which are mainly expressed on the lateral wall of OHCs and the glucose antagonist and ATP regulate this energy transport mechanism. S. Liu et al. (“The Key Transcription Factor Expression in the Developing Vestibular and Auditory Sensory Organs: A Comprehensive Comparison of Spatial and Temporal Patterns”) determine that Pax2, Sox2, and Prox1 have differential and overlapping temporospatial expression patterns during the development of vestibular and auditory sensory organs in mice.

## 2. Hair Cell Damage and Protection

B. Tong et al. (“Mechanisms of Hearing Loss in a Guinea Pig Model of Superior Semicircular Canal Dehiscence”) explore the pathogenesis of superior semicircular canal dehiscence (SSCD) in the guinea pig model and report that the bony fenestration of the superior semicircular canal mimics the hearing loss pattern of SSCD patients. J. Hong et al. (“N-Methyl-D-Aspartate Receptors Involvement in the Gentamicin-Induced Hearing Loss and Pathological Changes of Ribbon Synapse in the Mouse Cochlear Inner Hair Cells”) investigate that the N-methyl-D-aspartate receptors regulate the number and distribution of inner HC ribbon synapses after gentamicin-induced ototoxicity and their inhibition by antagonist minimized the drug-induced ototoxicity, and thus maintain the integrity of ribbon synapses. L. Xia et al. (“Comparison of Acceptable Noise Level Generated Using Different Transducers and Response Modes”) compare the acceptable noise level (ANL) in 20 mandarin subjects with normal hearing. The author obtained ANL through different methods and determined that the ANL in normal hearing listeners may not be affected by different modes of presentation. M. Waqas et al. (“Inner Ear Hair Cell Protection in Mammals against the Noise-Induced Cochlear Damage”) provide a brief review about the mechanisms involved in the HC loss after noise-induced trauma and discuss the recent HC protection strategies to prevent and recover hearing function in mammals after noise-induced damage. X. Cheng et al. (“The Benefits of Residual Hair Cell Function for Speech and Music Perception in Pediatric Bimodal Cochlear Implant Listeners”) report that the combination of electric and acoustic hearing significantly improves the perception of music and Mandarin tones in pediatric cochlear implant patients. X. Ding et al. (“The Characteristic and Short-Term Prognosis of Tinnitus Associated with Sudden Sensorineural Hearing Loss”) determine the association between tinnitus and sudden sensorineural hearing loss (SSNHL). The authors found that tinnitus can be ameliorated by the successful treatment of SSNHL. G. Li et al. (“The Role of Autoimmunity in the Pathogenesis of Sudden Sensorineural Hearing Loss”) provide a brief review on the autoimmune mechanisms involved in SSHL and discuss the role of immunosuppressive drugs in immune therapy. N. Zhao et al. (“Functional Change in the Caudal Pontine Reticular Nucleus Induced by Age-Related Hearing Loss”) report that the age-related hearing loss causes an increase in PnC sensitivity that in turn enhances acoustic startle responses in C57 mice. B. Li et al. (“Effects of Various Extents of High-Frequency Hearing Loss on Speech Recognition and Gap Detection at Low Frequencies in Patients with Sensorineural Hearing Loss”) investigate the extent of SNHL at high frequencies influences the ability to recognize compressed speech of lower frequencies in hearing loss patients.

## 3. Hair Cell Regeneration

M. Tang et al. (“Potential Application of Electrical Stimulation in Stem Cell-Based Treatment against Hearing Loss”) provide a comprehensive review to address the current challenges and problems in stem cell transplant-based treatments in the inner ear against deafness and present a critical viewpoint about electrical stimulations as a physical factor to modulate stem cell behavior and promote stem cell therapy to treat hearing loss.

## 4. Spiral Ganglion Neuron Development and Protection

J. Li et al. (“Contralateral Suppression of DPOAEs in Mice after Ouabain Treatment”) determine that the type II SGNs participate in the contralateral suppression of the medial olivocochlear reflex after selectively inducing apoptosis in the type I SGNs using ouabain treatment.

## 5. Inherited Hearing Loss

H. Du et al. (“Identification of Binding Partners of Deafness-Related Protein PDZD7”) determine the eleven novel PDZD7-binding proteins through yeast two-hybrid screening that are expressed in the inner ear. Most of the new PDZD7-binding partners such as TRIM35, CADM1, AMOT, BLZF1, Numb, KCDT10, CCDC27, and TRIP11 have not been reported before and will help to understand the role of PDZD7 in hearing transduction. P. Li et al. (“Knock-In Mice with Myo3a Y137C Mutation Displayed Progressive Hearing Loss and Hair Cell Degeneration in the Inner Ear”) report that Myo3a kinase domain Y137C mutant mice have an elevated hearing threshold, degenerated inner ear HCs, and structural abnormality in HCs stereocilia after 6 months of age, thus Myo3a is essential for maintaining the intact structure of HC and normal hearing function. S. Hu et al. (“Genetic Etiology Study of Ten Chinese Families with Nonsyndromic Hearing Loss”) identify novel pathogenic variants in six Chinese families with a hereditary hearing loss by targeted next generation sequencing. F. Zhang et al. (“Three *MYO15A* Mutations Identified in One Chinese Family with Autosomal Recessive Nonsyndromic Hearing Loss”) report three MYO15A variants c.3971C>A (p.A1324D), c.4011insA (p.Q1337Qfs^∗^22), and c.9690+1G>A. These variants are absent in 200 normal controls and cosegregated with hearing disability in this family. X. Wang et al. (“A Novel p.G141R Mutation in *ILDR1* Leads to Recessive Nonsyndromic Deafness DFNB42 in Two Chinese Han Families”) identify a novel p.G141R homozygous mutation in ILDR1 gene that may be the genetic cause of deafness in two unrelated Chinese Han families. X. Wu et al. (“Autosomal Recessive Congenital Sensorineural Hearing Loss Due to a Novel Compound Heterozygous PTPRQ Mutation in a Chinese Family”) report that the novel compound heterozygous missense mutation c.4472C>T p.T1491M and c.1973T>C p.V658A in PTPRQ gene is the genetic cause of recessively inherited sensorineural hearing loss in a Chinese family.

## 6. Inner Ear Drug Delivery System

X. Xu et al. (“Hollow Mesoporous Silica@Zeolitic Imidazolate Framework Capsules and Their Applications for Gentamicin Delivery”) synthesize a new nanoparticle capsule that can be used as a drug delivery route for the gentamicin transfer at the specific site in the inner ear. The authors also determine the sustained release capacity of gentamicin from the capsule by *in vitro* and *in vivo* assays.

We believe that the studies included in this second special issue of “Hearing Loss: Reestablish the Neural Plasticity in Regenerated Spiral Ganglion Neurons and Sensory Hair Cells” provide important insights into cochlear physiology and pathology as well as the important progress in technology that can be translated into clinical application of the medical treatment of cochlear damage in SNHL. We wish that this special issue will represent a significant contribution in the effort to achieve effective protection and treatment of hearing loss in the near future.

